# Incidence of cleft-related speech problems in children with an isolated cleft lip

**DOI:** 10.1007/s00784-020-03367-5

**Published:** 2020-06-04

**Authors:** B. J. A. Smarius, S. Haverkamp, H. de Wilde, A. van Wijck-Warnaar, A. B. Mink van der Molen, C. C. Breugem

**Affiliations:** 1 Department of Pediatric Plastic Surgery, Wilhelmina Children’s Hospital, University Medical Center Utrecht, University of Utrecht, Heidelberglaan 100, P.O. BOX 85500, 3508 GA Utrecht, The Netherlands; 2 Department of Surgery, Meander Medical Centre, Amersfoort, The Netherlands; 3 Speech and Language Therapy, Wilhelmina Children’s Hospital, University Medical Center Utrecht, Utrecht, The Netherlands; 4 Department of Otorhinolaryngology-Head and Neck Surgery, University Medical Center Utrecht, Utrecht, The Netherlands; 5 Department of Plastic Surgery, St. Antonius Hospital, Nieuwegein, The Netherlands; 6 Department of Plastic Surgery, Meander Medical Center, Amersfoort, The Netherlands; 7 Department of Plastic Surgery, Emma Children’s Hospital, University Medical Center Amsterdam, Amsterdam, The Netherlands

**Keywords:** Isolated cleft lip, Cleft lip, Cleft-related speech problem, Speech therapy, AOM/OME

## Abstract

**Objectives:**

Clinicians agree that children with isolated cleft lip have fewer cleft-associated problems than children with cleft lip and palate. Unfortunately, for isolated cleft lip children, the risk of cleft-associated problems is unknown and maybe underestimated. Often, these children do not get the required follow-up by a multidisciplinary team and thereby not the known benefits in supporting their development. This study examines the incidence of cleft-related speech problems and ear problems in children with isolated cleft lip.

**Materials and methods:**

A prospective study was performed on all children born with an isolated cleft lip and treated at the Wilhelmina Children’s Hospital in Utrecht between January 2007 and April 2014. Data were collected for sex, date of birth, genetics, cleft lip type, date of cleft lip repair, type of repair, speech/language problems, and ear problems.

**Results:**

This study included 75 patients (59% male). The mean age of the children at the moment of speech examination was 32.5 months (SD 6.1). Eighteen of the 75 children (24%) needed speech and language therapy; however, only one child (1.3%) had a cleft-related speech problem. Sixteen of the 75 patients (21%) reported a history of one or more episodes of acute otitis media (AOM)/otitis media with effusion (OME) during the first 6 years.

**Conclusion/clinical relevance:**

This is the first prospective study analyzing the incidence of cleft-related speech problems in children with an isolated cleft lip. These children do not have a higher risk of cleft-related speech problems or AOM/OME when compared to the general population. However, children with an isolated cleft do have a higher incidence of speech therapy.

**Electronic supplementary material:**

The online version of this article (10.1007/s00784-020-03367-5) contains supplementary material, which is available to authorized users.

## Introduction

The incidence of cleft lip/palate in the Netherlands ranges from 1.4 to 2.1 per 1000 [1]. The inability to close the nasopharynx in case the palate is affected often results in problems with feeding, hearing, speech, and language development [2–4].

Clinicians involved in the care of children with orofacial clefts agree that children with isolated cleft lip have fewer cleft-associated speech and ear problems than children with cleft lip and palate [5]. In most treatment protocols, children with cleft lip and palate are analyzed in a multidisciplinary team. Unfortunately, for children with an isolated cleft lip, the risk of cleft-associated speech and ear problems is unknown and maybe underestimated. It is possible these children do not get the required follow-up by a multidisciplinary team and thereby not the known benefits in supporting their development.

Cleft-related speech problems should be distinguished from non-cleft-related speech problems. Cleft palate patient may be at risk of developing certain deviant speech characteristics, affecting resonance, articulation, and intelligibility, directly caused by the original anomaly and/ or related to incompetent velopharyngeal function.

Velopharyngeal insufficiency (VPI) plays an important role in cleft-related speech problems. VPI is a term used to describe disorders characterized by the abnormal function of the velopharyngeal valve. When the velopharyngeal valve is not functioning correctly, it can cause complications with speech. This can result in several speech disorders, such as hypernasal speech, inability to generate pressure for speech sounds, or the inability to form speech sounds correctly.

Also, the occurrence of recurrent acute otitis media (AOM) and otitis media with effusion (OME) has been reported to be higher in children with cleft palate [6, 7]. The etiologic basis for middle ear pathology and hearing loss in patients with cleft palate is considered to be Eustachian tube dysfunction due to functional obstruction, secondary to failure of the palatal muscles to assist in opening the Eustachian tube [8]. Hearing loss in early childhood associated with otitis media with effusion may result in impaired speech, language, and even cognitive development [9].

Vallino et al. concluded that children with isolated cleft lip who develop cleft-associated problems need to be monitored by the multidisciplinary team until all management needs are reached [5].

A multicenter questionnaire study in our hospital investigated the isolated cleft lip population [10]. This retrospective study concluded that isolated cleft lip patients often receive speech and language therapy and these patients should be investigated by the cleft palate team because of the high risk of cleft palate-related symptoms.

Subsequently, since that study, all patients with an isolated cleft lip were prospectively analyzed at the age of 3 years by the cleft team of the University Medical Centre Utrecht (UMCU), the Netherlands.

This study examines the incidence of cleft-related speech and ear problems in children with isolated cleft lip. The study is done to assess whether follow-up is needed in children with isolated cleft lip and gives recommendations regarding follow-up within a team of this specific subgroup of cleft patients.

## Methods

Permission for this study was obtained from the Medical Ethics Review Committee (METC) Board at the University Medical Center, Utrecht, the Netherlands (reference number WAG/mb/17/032751).

All children born with an isolated cleft lip (with or without involvement of the alveolus) and treated at the Wilhelmina Children’s Hospital between January 2007 and April 2014 were eligible for this prospective study. All children with any cleft palate involvement were excluded. Data were collected for sex, date of birth, cleft lip type, possible syndromes, date of cleft lip repair, type of repair, speech and language problems, and ear problems.

### Definition of isolated cleft lip

An isolated cleft lip is a cleft that involves the lip only without involvement of the palate. Isolated cleft lip may exist with or without any (complete or incomplete) alveolar cleft. If these structures are partially involved, it is referred to as an incomplete cleft lip. Cleft lip can occur as a one sided (unilateral) or two sided (bilateral). The definition of a cleft lip in this study is an isolated cleft lip with or without involvement of the alveolus.

### Cleft lip/palate team care

It is the standard care at the Wilhelmina Children’s Hospital for a specialized multidisciplinary cleft team to analyze all children with all cleft types. Children undergo assessments with a speech and language pathologist, otolaryngologist, pediatric dentist, and plastic surgeon. At the age of 3 years, a speech assessment is a component of the cleft lip/palate program follow-up for isolated cleft lip patients since 2007.

### Oral assessment

At the age of 3 years, the plastic surgeon excluded a cleft palate by visual inspection and if necessary by palpating a possible submucous cleft.

### Speech and language assessment

The speech therapists who work in our cleft team serially assess the speech of patients with a cleft or velopharyngeal dysfunction with other etiology. In the Netherlands, cleft palate patients speech is assessed according The Dutch Cleft Speech Evaluation Test (DCSET) designed for children with orofacial clefts (Supplemental Table: DCSET) [11]. This test has been implemented nationwide in the Netherlands. Speech characteristics in the DCSET are evaluated by the speech therapist of our team. The speech therapists in Utrecht do participate in the national calibration sessions for the DCSET.

In our center, the speech therapists assessed all children with isolated cleft lip at the age of 3 years old using the DCSET. Speech was assessed in the following order: resonance, nasal emissions, oral facial muscle function, intelligibility, articulation, and consonant production.

The resonance was subjectively evaluated while the patient speaks loudly 6 nasal, 5 oronasal, and 6 oral sentences. Resonance was sored for each sentence on a 3-point scale. A score of 1 was given for normal resonance, and a score of 3 for severe hypernasality or hyponasality. Nasometry was not used to analyze the resonance because of the insufficient cooperation with the nasometer in young children (< 4 years) [12].

Mirror test were performed to detect nasal emissions.

Orofacial muscle function was observed during the assessment. Attention was paid to the following: open mouth, tongue position, and mouth breathing.

**Fig. 1:**
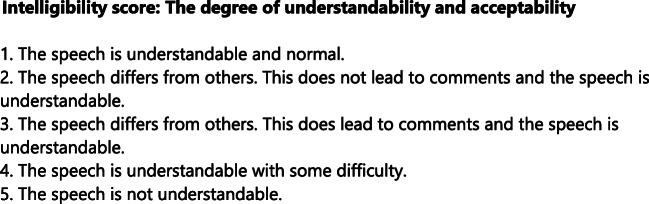
Intelligibility score: the degree of understandability and acceptability. 1. The speech is understandable and normal. 2. The speech differs from others. This does not lead to comments and the speech is understandable. 3. The speech differs from others. This does lead to comments and the speech is understandable. 4. The speech is understandable with some difficulty. 5. The speech is not understandable

The intelligibility was sored during spontaneous speech. Intelligibility was scored on a 5-point scale. A description of the intelligibility scores used by the parents and speech pathologists is presented in Fig. 1.

Finally, articulation was evaluated. Patients were asked to speak aloud words and sentences in a playful way, depending on the age of the patient. If a misarticulation occurred, the type of error was indicated on the form.

### Acute otitis media (AOM) and otitis media with effusion (OME)

AOM is one of the most common infections in (early) childhood. It is defined as the presence of middle-ear effusion in conjunction with rapid onset of one or more signs or symptoms of inflammation of the middle ear such as fever, otalgia, and ear discharge (otorrhoea). Middle-ear effusion without signs of an acute infection indicates otitis media with effusion (OME or “glue ear”) [13]. The presence or absence of AOM/OME was determined and documented by the pediatric otolaryngologist. Otitis media with middle ear effusion with consequent conductive hearing loss was an indication for insertion of ventilation tubes. The number of episodes of AOM/OME and insertion of ventilation tubes was evaluated. If there was a clinical suspicion on hearing loss, the pediatric audiologist conducted hearing tests using an audiometer.

### Operation technique

In this study, all cleft lip surgeries were performed according to the standard treatment protocol at our department and were performed under general anesthesia by two experienced cleft surgeons. Children underwent cleft lip repairs according to Fisher, Tennison, or Mulliken (in bilateral cleft lip) at the age of 3 to 6 months [14–16]. Using these techniques, the cleft repair is combined with paranasal and perioral muscle reconstruction.

### Analysis

Patient characteristics were summarized by descriptive statistics. The Wilcoxon signed-rank test was used for data analysis of the intelligibility scores. All statistical analyses were performed using IBM Statistical Package for Social Science (SPSS) version 22 (SPSS Inc., Chicago, IL, USA). All calculated *P* values were considered significant if less than 0.05.

## Results

### Characteristics

This study included 85 patients who underwent only a cleft lip operation between January 2007 and April 2014. No submucous cleft was identified in this subgroup of patients. Ten patients were lost to follow-up or had incomplete follow-up information. A total of 75 children had complete analyses and were included for analyses. Fifty-nine percent (*n* = 44) of the children were boys. Mean age at cleft lip surgery was 3.6 months (SD 1.1). Patient characteristics are listed in Table 1.

**Table 1:** Patient characteristics

Characteristics	Patients
	*n* = 75(%)
Gender	
Male	44 (59)
Female	31 (41)
Age	
Mean age at cleft lip repair	3.6 months (SD 1.1)
Mean age at speech assessment	32.5 months (SD 6.1)
Cleft lip type	
Incomplete unilateral	38 (51)
Incomplete unilateral + alveolus involvement	19 (25)
Complete unilateral	1 (1)
Complete unilateral + alveolus involvement	15 (20)
Complete bilateral	1 (1)
Complete bilateral + alveolus involvement	1 (1)

### Genetics and additional anomalies

Besides the thorough physical examination done in all children, genetic analysis was performed in 7% (*n* = 5) of the isolated cleft lip patients. An overview of additional anomalies can be found in Table 2.

**Table 2:** Patient characteristics

Functional heart murmur (*n* = 5)	
Inguinal hernia (*n* = 2)	
Psychomotor retardation (*n* = 2)	
Undescended testis (*n* = 1)	
Hip dysplasia (*n* = 1)	
Down syndrome/ASD (*n* = 1)	
Recurrent intussusception (*n* = 1)	
Congenital atresia of the ear (*n* = 1)	

### Isolated cleft lip type

Seventy-six percent of the children (*n* = 57) had an incomplete cleft lip. Ninety-eight percent of the children (*n* = 73) had a unilateral cleft. Further information of the group is listed in Table 1.

### Technique cleft lip closure

Eighty-three percent of the children were operated according to Fisher (*n* = 62), 15% according to Tennison (*n* = 11), and 3% according to Mulliken (*n* = 2).

### Speech and language assessment

The mean age of the children at the moment of speech examination was 32.5 months (SD 6.1). Eighteen of 75 children (24%) noticed speech and language problems during the assessment. All those 18 children received speech and language therapy. Three of those 18 children with speech problems had an additional congenital syndrome that most likely influenced the speech development. Exclusion of those 3 children gives an indication for speech and language therapy in 21% of the children (15/72). Table 3 shows the frequency of occurrence for the features related to speech, language, and hearing in children who needed speech and language therapy.

**Table 3:** The frequency of occurrence for the features related to speech, language, and hearing in children who needed speech and language therapy

Patient	Male/female	Age***	Cleft lip type	Involvement alveolus	Anomalies	Resonance	Nasal emission	Orofacial muscle function	Articulation	Intelligibility score speech therapist	Intelligibility score parent	Cleft-related speech problem	AOM/OME
1	M	33	Incomplete unilateral	−	−	−	−	−	+	3−4	3−4	−	+
2	M	37	Incomplete unilateral	−	−	−	−	+	−	1	1	−	−
3	F	32	Incomplete unilateral	−	Psychomotor retardation	−	−	+	+	3	4	−	+
4	F	22	Complete unilateral	+	−	−	−	+	+	2	2	−	−
5	M	34	Incomplete unilateral	−	−	−	−	+	+	2	2	−	+
6	F	23	Incomplete unilateral	+	−	−	−	+	+	2	2	−	−
7	M	43	Incomplete unilateral	−	−	−	−	−	+	2	2	−	−
8	F	34	Incomplete unilateral	+	Psychomotor retardation	−	−	−	+	1−2	2	−	−
9	M	33	Incomplete unilateral	+	−	−	−	+	−	1	1	−	+
10	M	25	Incomplete unilateral	−	−	−	−	+	+	2	2	−	+
11	M	27	Incomplete unilateral	+	−	−	−	+	+	4	4	−	−
12	M	34	Complete unilateral	+	−	−	−	+	+	2	2−3	−	+
13	M	34	Complete unilateral	+	−	−	−	−	+	3	2−3	−	−
14 *	F	41	Complete unilateral	+	−	−	−	−	+	1−2	2	+	−
15	M	35	Incomplete unilateral	+	Down syndrome	−	−	+	−	4−5	4−5	−	−
16	M	35	Incomplete unilateral	−	−	−	−	−	+	2−3	4	−	−
17 **	M	22	Incomplete unilateral	−	−	−	−	−	−	1	1	−	−
18 **	M	35	Incomplete unilateral	−	−	−	−	−	−	1	1	−	−

*AOM/OME* acute otitis media (AOM) or otitis media with effusion (OME)

*Mild cleft-related speech problem: interdental speech, palatalization, assimilation

**Speech therapy because of language delay

***Age at moment of speech assessment

All 18 patients scored a normal resonance during assessment.

There was no nasal emission in one of the 18 patients using the mirror test.

In 10 of the 75 patients (13%), abnormal orofacial muscle function was observed. In all cases, there was open mouth behavior, which was an indication to start speech and language therapy.

The intelligibility scores evaluated by the speech pathologist and the parents are presented in Table 3. The mean level of intelligibility was 2.2 (range 1–5) and 2.3 (range 1–5) as evaluated by the speech pathologist and parents, respectively. There was no significant difference (*P* = 0.084) in intelligibility score between the speech pathologist and parents, 2.2 vs 2.3 respectively.

The presence of articulation errors was documented in 13 of the 75 patients (17%) who needed speech and language therapy. In 1 child (1/75; 1.3%), there was a mild cleft-associated speech problem; this was expressed by interdental speech, palatalization, and assimilation. This patient was treated well with speech and language therapy. A possible submucous cleft was excluded as far as possible by palpating the palate.

### Ear problems (AOM/OME)

Sixteen (21%) of the 75 patients reported an onset of AOM/OME. Most children (63%) reported an onset of AOM/OME between the ages of 0 and 3 years. Sixty-nine percent (*n* = 11) of the children reported a history of 1–3 episodes of AOM/OME during the first 6 years, while 5 patients had 4 or more episodes (Table 4).

**Table 4:** Acute otitis media (AOM)/ Otitis media with effusion (OME) and ventilation tubes

	Total AOM/OME
	*N* (%)
Number of period AOM/OME	16
1 time	7 (44)
2–3 times	4 (25)
4–5 times	2 (13)
> 5 times	3 (19
Age of onset AOM/OME	
< 1 year	2 (13)
1–3 years	8 (50)
4–6 years	5 (31)
> 6 years	1 (6)
Number of insertion VT	
0 time	5 (31)
1 time	8 (50)
2–3 times	3 (19)
Age of onset VT	
< 1 year	0 (0)
1–3 years	9 (56)
4–6 years	2 (13)
> 6 years	0 (0)

*VT* ventilation tubes

Six of the 16 children (38%) with a history of AOM/OME received speech and language therapy. Three of those 6 children had 2 or more episodes of AOM/OME.

Eleven children (69%) underwent insertion of ventilation tubes because of consequent conductive hearing loss. Three patients underwent two or more ventilation tube insertions (Table 4).

In 1 child, there was a hearing loss as the result of unilateral congenital atresia of the ear; however, there was normal speech development.

### Secondary lip surgery

In 1 patient (1.3%), secondary lip surgery was required because of inadequate lip movements. Eleven patients (14.7%) underwent secondary lip surgery because of cosmetic concerns. All 12 patients who underwent a secondary lip surgery had a unilateral cleft lip. Eleven of the 12 children were treated according to Fisher during primary lip closure. The mean age at secondary cleft lip surgery was 42 months (range 17–79 months).

## Discussion

The primary objective in the surgical repair of a cleft lip and/or palate is to achieve an anatomical palatal closure with normal development of speech, hearing, and feeding, while minimizing the possible negative effect of surgery on maxillary outgrowth [17, 18]. Isolated cleft lip patients have fewer cleft-associated problems than patients with cleft palate involvement [5]. This study provides insights in the development of cleft-associated problems (speech and hearing) in children with isolated cleft lip.

In this study, 24% of the children needed speech and language therapy. However, 16% (12/75) of the children were recorded as having not cleft-associated articulation problems and 0% had problems with resonance. One child (1.3%) was associated with a cleft related articulation problem. Studies of the last 10 years showed comparable outcomes in isolated cleft lip patients (Table 5) [5, 10, 19–27]. However, the reported not cleft-associated speech disorders in this study was higher than in the general population (1–12%) (Table 5). This can be possible explained by the “extra” follow-up of a multidisciplinary team and the parents’ concern in children with an isolated cleft lip. Due to the fact that much attention is paid to the speech development, minor deviations are quickly noticed. Perhaps, those isolated cleft lip children will be referred to a speech and language pathologist more easily/at a lower threshold than children in the general population.

**Table 5:** Prevalence estimates of articulation and language disorders in preschool children

Study	Population	Age	% articulation and language disorders	Cleft-related speech problems
Present study (Smarius et al)	Cleft lip only	3 years	Articulation 17% Speech and language therapy 20%	1.3%
Vallino et al. [5]	Cleft lip only	3 years	Articulation 13% Language 18%	Not mentioned
Deelder et al. (2009)	Cleft lip only	0.3 to 13.1 years	Articulation 15%/22% Language 8%	Not mentioned
Tenenbaum [19]	Cleft lip only	3 to 17 years	Articulation and language 34%	Not mentioned
Witt et al. [20]	All clefts	–	VPI after operation 23%	23%
Bicknell et al. [21]	All clefts	–	VPI after operation 25%	25%
Mahoney et al. [22]	All clefts	–	VPI after operation 10.3%	10.3%
Stevenson and Richman [23]	General population	3 years	Language 3.1%	–
Silva [24]	General population	3 years	Language 8.4%	–
National Institute of Deafness and other communication disorders [25]	General population	5–8 years	Speech sound disorder 8–9%	–
Law et al. [26]	General population	2–5 years	Language 5–12%	–
Hannus et al. [27]	General population	< 6 years	Language 10 per 1.000	–

In this study, 1 child (1.3%) was associated with a cleft-related speech problem. There are few studies of articulation of children with isolated cleft lip. Riski and DeLong (1984) established that children with isolated cleft lip developed articulation skills that followed a normal developmental schedule [28]. Kono et al. [29] reported that 5% of the 71 children with cleft lip had velopharyngeal inadequacy and no other visible palatal anomaly [29]. To compare with other clefts, 10–30% of the primary cleft palate closures still have velopharyngeal insufficiency, and secondary surgery is often imperative [20–22].

Our retrospective study from Deelder et al. concluded that isolated cleft lip patients often receive speech and language therapy compared to the general population [10]. Because of its retrospective nature, we started this prospective study. After this prospective study, we have the same conclusion, but with a 1.3% cleft-associated speech problem, it seems that isolated cleft lip patients do not have more risk to develop possible velopharyngeal insufficiency than the general population without a cleft lip and/or palate.

Gosain et al. reported “some form of submucous cleft palate” in 36% of the cleft lip patients. A submucous cleft palate may go unnoticed in isolated cleft lip patients [30]. This could be a reason for those patients to develop cleft-associated problems. All patients in our group had a submucous cleft excluded with physical examination; however, we agree with Gosain et al. to screen all isolated cleft lip patients for a possible submucous cleft palate [30]. Since we had only 1 child with mild VPI that was treated well with speech therapy, we could not demonstrate the suggestion that an associated submucous cleft is common in isolated cleft lip patients.

Recently, Ruegg et al. demonstrated that in non-syndromic CL patients, 31% had chronic middle ear infections compared to 11% in the control group [31]. They suggest that an abnormal musculature (missed submucous cleft) could be the cause. In the absence of a cleft palate, the alteration of the shape and size of soft palate, the Eustachian tube itself, the cranial base, or the nasopharyngeal space, could be the cause. However, Ruegg et al. only send questionnaires to parents, while the patients in our group had a physical investigation [31].

Otitis media with effusion (OME) is fluid in the middle ear without signs or symptoms of inflammation. Up to 80% of children have been affected by OME by the age of 4 years [32]. Acute otitis media (AOM) is characterized by the presence of middle-ear effusion together with an acute onset of signs and symptoms caused by middle-ear inflammation [13]. In high-income countries, the incidence of AOM in children aged 0–5 years ranges from 136 to 273 per 1000 child-years with a peak incidence in the first 2 years of life [33–36]. In the Dutch study from De Hoog et al., 39% experienced a first AOM before 2 years in the general population [37]. In our study, 21% (16/75) of the isolated cleft lip children in this study reported a history of one or more episodes of AOM/OME during the first 6 years. This is comparable to the 30% AOM in the study of Deelder et al. [10]. Our study showed a low prevalence of AOM/OME compared to the general population in earlier studies [33–37]. We can conclude that there is no increased risk of AOM in our study population (isolated cleft lip) compared to general population. Thereby, no extra otologic follow-up should be provided to children with isolated cleft lip to prevent hearing loss as a result of recurrent otitis media.

Internationally, the use of different scales to measure speech parameters impedes comparisons of outcomes following treatment [38]. In the Netherlands, the speech of cleft palate patients is assessed according DCSET designed for children with orofacial clefts. For the assessment of the speech in this study, the speech and language pathologist used a subtest of the DCSET because of the young age of the patients (mean age 32.5, SD 6.1). Spruijt et al. measured the inter- and intra-rater reliability of the DCSET providing the possibility of benchmarking the outcome from Dutch cleft lip and palate teams with results from abroad [11]. They concluded that the strength of intra- and inter-rater agreement for most of the parameters was good or very good.

The strength of this study includes the fact that all the patients were operated by two surgeons with a comparable technique and standardized prospective postoperative care and follow-up. All patients with an isolated cleft lip in our hospital are followed up and analyzed at the age of 3 years by the cleft team, and special oral investigations were performed to make sure that no submucous cleft is missed.

In conclusion, this is the first prospective study analyzing the incidence of cleft-related speech problem in children with an isolated cleft lip. These children do not have a higher risk of cleft-related speech problems and AOM/OME than non-cleft children; however, patients with an isolated cleft lip often receive speech and language therapy. We recommend after this study to follow-up isolated cleft lip patients according the local cleft palate protocol but—based on this study—there seems to be no indication for a speech and ear assessment at the age of 3 years old by the speech therapist and otolaryngologist.

## Electronic supplementary material

ESM 1 (DOCX 23 kb)
