# Supplementary material for: Incidence of cleft-related speech problems in children with an isolated cleft lip
Source: Clin Oral Investig. 2020 Jun 4;25(3):823–31. doi: 10.1007/s00784-020-03367-5 (PMC7878222; doi:10.1007/s00784-020-03367-5)
Supplement: Supplementary file 1 — (DOCX 23 kb) [file 784_2020_3367_MOESM1_ESM.docx]

**Subtest of the Dutch Cleft Speech Evaluation Test (DCEST)**

Test form for evaluation of speech

- 1. *Nasal resonance (subjectively)*
  2. *Mirror test*
  3. *Orofacial muscle function*
  4. *Intelligibility*
  5. *Articulation*

**A. Assessment of nasal resonance**

Perceptual scale nasal resonance: score 1, 2, or 3

Nasometer, from age 5 years: not done in this study

*Nasal passage:*

| Sentences |  | Hypernasal  resonance (1 - 3),  percentage | Hyponasal  resonance (1 - 3),  percentage |
| --- | --- | --- | --- |
| 1 | Mama gaat naar oma |  |  |
| 2 | Zij neemt een mand mee |  |  |
| 3 | In de mand zijn bananen |  |  |
| 4 | En ook mandarijnen |  |  |
| 5 | Oma heeft honger |  |  |
| 6 | Zij neemt een mandarijn |  |  |

*Mixed passage*

| Sentences |  | Hypernasal  resonance (1 - 3),  percentage |
| --- | --- | --- |
| 1 | Miep is op school |  |
| 2 | Nu gaat zij kleuren |  |
| 3 | Zij tekent de juf |  |
| 4 | Dat wordt heel mooi |  |
| 5 | Juf geeft Miep stickers |  |

*Denasal passage:*

| Sentences |  | Hypernasal  resonance (1 - 3),  percentage |
| --- | --- | --- |
| 1 | Jos heeft feest |  |
| 2 | Hij is jarig |  |
| 3 | Hij krijgt veel kadootjes |  |
| 4 | Ook is er taart |  |
| 5 | De taart heeft vijf kaarsjes |  |
| 6 | Jos blaast ze uit |  |

**B. Mirror test**

- = the mirror does not fog.

+ = the mirror fogs.

|  | Right nostril | Left nostril | Audible | Turbulence |
| --- | --- | --- | --- | --- |
| Pa pa pa … |  |  |  |  |
| Pi pi pi … |  |  |  |  |
| Ka ka ka … |  |  |  |  |
| Ki ki ki … |  |  |  |  |
| Ssssssssssss |  |  |  |  |
| Ie……… |  |  |  |  |
| Oe…….. |  |  |  |  |
| Fffffffffffff |  |  |  |  |
| Piet zit op de stoep |  |  |  |  |

**C. Oral facial muscle function**

Attention was paid to:

- open mouth
- tongue position
- mouth breathing

**D. The degree of understandability and acceptability**

1. The speech is understandable and normal.

2. The speech differs from others. This does not lead to comments and the speech is understandable.

3. The speech differs from others. This does lead to comments and the speech is understandable.

4. The speech is understandable with some difficulty.

5. The speech is not understandable.

**E. Articulation assessment**

Patients were asked to speak aloud words and sentences, depends on the age of the patient in a playfull way.

1. Consonant production. Consonants and clusters in words.

| *Number* | *Consonant* | *Initial* | *Middle* | *Final* | *Error* |
| --- | --- | --- | --- | --- | --- |
| 1 | m | maan | emmer | boom |  |
| 2 | ng |  | vinger | ring |  |
| 3 | n | neus | banaan | pan |  |
| 4 | p | poes | appel | aap |  |
| 5 | t | tas | auto | bed |  |
| 6 | k | kam | beker | boek |  |
| 7 | d | dak | ladder |  |  |
| 8 | b | beer | glijbaan |  |  |
| 9 | f | fiets | tafel | dief |  |
| 10 | s | sok | vissen | muis |  |
| 11 | s+ptk | spin step |  | heks |  |
| 12 | s+ptk | spin step |  | heks |  |
| 13 | s+mn | smelt snor |  | spons |  |
| 14 | s+mn | smelt snor |  | spons |  |
| 15 | sj | jam | ijsje |  |  |
| 16 | g | gieter | ogen | oog |  |
| 17 | w | wiel | duwen |  |  |
| 18 | r | raam | toren |  |  |
| 19 | j | jas |  |  |  |
| 20 | l | lamp | molen | bal |  |
| 21 | n+t |  |  | hond |  |
| 22 | m+pt |  |  | lamp hemd |  |
| 23 | k+n | knopen |  |  |  |
| 24 | ng+kt |  |  | band springt |  |
| 25 | r+m |  |  | arm |  |
| 26 | l+m |  |  | helm |  |

1. Production of consonants and clusters in sentences

| *Number* | *Consonant* | *Sentence* | 1^st^ consonant | 2^nd^ consonant | *Error* |
| --- | --- | --- | --- | --- | --- |
| 1 | m | Oma mag zingen |  |  |  |
| 2 | ng | Een ring aan je vinger |  |  |  |
| 3 | n | Niels eet een banaan |  |  |  |
| 4 | p | Opa rookt een pijp |  |  |  |
| 5 | t | Ik heb twee toeters |  |  |  |
| 6 | k | Die kabouter lust geen melk |  |  |  |
| 7 | d | De dieven kijken in de doos |  |  |  |
| 8 | b | De baby blijft lang huilen |  |  |  |
| 9 | f | Geef me de voetbal |  |  |  |
| 10 | s | Sanne snoept van de smeerkaas |  |  |  |
| 11 | s+ptk | Saskia speelt poppenkast |  |  |  |
| 12 | s+ptk | Saskia speelt poppenkast |  |  |  |
| 13 | s+mn | Sanne snoept van de smeerkaas |  |  |  |
| 14 | s+mn | Sanne snoept van de smeerkaas |  |  |  |
| 15 | sj | Sjors eet een ijsje |  |  |  |
| 16 | g | Gijs heeft een spiegel |  |  |  |
| 17 | w | Hij zwemt in het water |  |  |  |
| 18 | r | Wij horen muziek op de radio |  |  |  |
| 19 | j | Joop gaat gras maaien |  |  |  |
| 20 | l | De baby blijft lang huilen |  |  |  |
| 21 | n+t | Tante Tine heeft een hond |  |  |  |
| 22 | m+pt | Remco loopt op klompen |  |  |  |
| 23 | k+n | Zij knipt de knopen van de jas |  |  |  |
| 24 | ng+kt | Wim springt op de bank |  |  |  |
| 25 | r+m | De arme man heeft het warm |  |  |  |
| 26 | l+m | Helma eet graag zalm |  |  |  |
